# Prospective evaluation of circumferential and longitudinal strain in asymptomatic children with dual ventricles who underwent single ventricle repair: comparison to single LV, single RV and normal hearts

**DOI:** 10.1186/1532-429X-17-S1-P212

**Published:** 2015-02-03

**Authors:** Ramkumar Krishnamurthy, Cory V Noel, Amol Pednekar, Ricardo Pignatelli, Rajesh Krishnamurthy

**Affiliations:** Pediatric Cardiology, Baylor College of Medicine, Houston, TX USA; Radiology, Texas Children’s Hospital, Houston, TX USA; Clinical Science, Philips Healthcare, Houton, TX USA

## Background

Rarely, patients with normally sized RV and LV will undergo total cavopulmonary connection (TCPC) due to the complexity of their intracardiac anatomy giving them a dual ventricle (DV) for a single cardiac output. The ventricular function in this unique physiology compared to SRV, SLV and normal hearts remains poorly understood, with few studies performed^1-3^. In this study, we perform a comprehensive comparison of global and regional strain in both the circumferential (ε_cc_) and longitudinal (ε_L_) dimensions to conventional SV hearts and normal hearts.

## Purpose

In normal subjects and asymptomatic patients with DV (LV and RV calculated independently), SLV and SRV after TCPC, to compare:

1. Global ε_cc_ and ε_L_ strain,
2. Regional circumferential and longitudinal strains at free wall (ε_cc-free,_ ε_L-free_) and septum (ε_cc-sept,_ ε_L-sept_),
3. ε_cc_ and ε_L_ across the ventricle from apex to base.

## Methods

We performed a prospective analysis of 23 subjects (7 normals age in years: 11.8 +/- 3.1, 5 DV age: 12.4 +/- 2.7, 6 SRV age: 11.4 +/- 2.3, 5 SLV age: 12.6 +/- 4.2).

### Acquisition Protocol

Strain information was acquired at three short axis slices at basal, mid-cavity, and apical locations in all 123 subjects in a 1.5T MRI scanner (Philips Acheiva) using: a) Complementary Spatial Modulation of Magnetization (CSPAMM) images: Used for generating ε_cc_; and b) Fast-Strain Encoded (fSENC) images: Used for generating ε_L_.

### Data Analysis

ε_cc_ and ε_L_ were calculated from SAX slices using Diagnosoft^TM^_._ The ventricular regions at each slice were assigned based upon the AHA 16 segment model (fig. 1). ε_cc-sept,_ ε_L-sept_, ε_cc-free, and_ ε_L-free_ were also calculated for each slice and compared.

**Figure:**
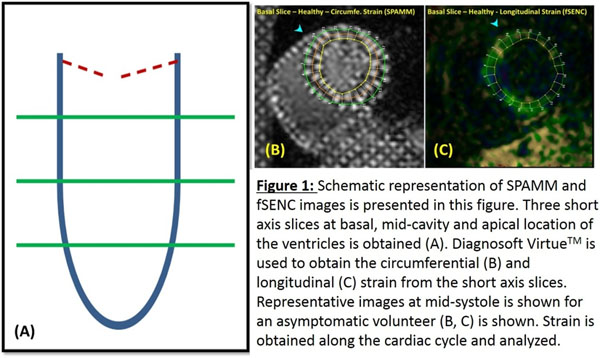
Figure 1

## Results

**Figure 2:**
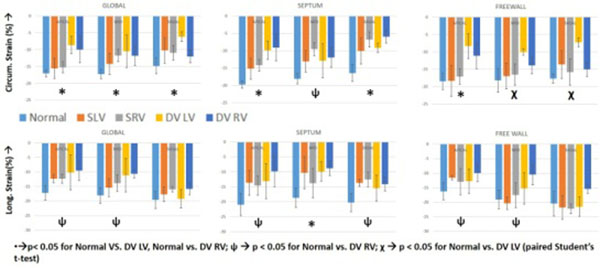
Bar plots comparing the longitudinal(ε_L_) and circumferential (ε_cc_) strain values in a pediatric population betweennormal subjects, patients with dual ventricles wih single ventricular repair and patients with systemic single ventricles. We demonstrate a significant reduction in both ε_L_ and ε_cc_ compared to normal.

1.) Compared to normals, there is a significant reduction in global ε_cc_ at all ventricular levels of DV patients (fig 2).

2.) Compared to normals, there is a significant reduction in global ε_L_ in mid-ventricular and apical locations of DV patients.

3.) The ε_cc_ of the LV of DV patients consistently lower than SLV for global and regional calculations.

4.) In the same DV patient at the basal location, the ε_cc-free_ was higher in the RV (75 +/- 42%), with the ε_L-free_ being higher in the LV (25 +/- 10%).

5.) Global ε_L_ progressed from base to apex in all groups.

## Conclusions

Strain values of the RV and LV in DV patients demonstrate significant differences compared to normal subjects. Additionally, the LV of DV patients had lower strain values than the SLV patients. The differences in the RV and LV within the same DV patient suggest inherent differences in ventricular biomechanics in this unique physiology. The shared workload of the LV and RV for a single cardiac output may contribute to their lower strain values.
